# Adhesion of *Crithidia fasciculata* promotes a rapid change in developmental fate driven by cAMP signaling

**DOI:** 10.1128/msphere.00617-24

**Published:** 2024-09-24

**Authors:** Shane Denecke, Madeline F. Malfara, Kelly R. Hodges, Nikki A. Holmes, Andre R. Williams, Julia H. Gallagher-Teske, Julia M. Pascarella, Abigail M. Daniels, Geert Jan Sterk, Rob Leurs, Gordon Ruthel, Rachel Hoang, Megan L. Povelones, Michael Povelones

**Affiliations:** 1 Department of Pathobiology, School of Veterinary Medicine, University of Pennsylvania, Philadelphia, Pennsylvania, USA; 2 Department of Biology, Villanova University, Villanova, Pennsylvania, USA; 3 Department of Biology, Haverford College, Haverford, Pennsylvania, USA; 4 Division of Medicinal Chemistry, Amsterdam Institute for Molecules, Medicines and Systems, Faculty of Science, Vrije Universiteit Amsterdam, De Boelelaan HZ, Amsterdam, the Netherlands; Cleveland State University, Cleveland, Ohio, USA

**Keywords:** *Crithidia fasciculata*, adhesion, attachment, cyclic AMP, signaling, differentiation, kinetoplastid

## Abstract

Trypanosomatids are single-celled parasites responsible for human and animal disease. Typically, colonization of an insect host is required for transmission. Stable attachment of parasites to insect tissues *via* their single flagellum coincides with differentiation and morphological changes. Although attachment is a conserved stage in trypanosomatid life cycles, the molecular mechanisms are not well understood. To study this process, we elaborate upon an *in vitro* model in which the swimming form of the trypanosomatid *Crithidia fasciculata* rapidly differentiates following adhesion to artificial substrates. Live imaging of cells transitioning from swimming to attached shows parasites undergoing a defined sequence of events, including an initial adhesion near the base of the flagellum immediately followed by flagellar shortening, cell rounding, and the formation of a hemidesmosome-like attachment plaque between the tip of the shortened flagellum and the substrate. Quantitative proteomics of swimming versus attached parasites suggests differential regulation of cyclic adenosine monophosphate (cAMP)-based signaling proteins. We have localized two of these proteins to the flagellum of swimming *C. fasciculata*; however, both are absent from the shortened flagellum of attached cells. Pharmacological inhibition of cAMP phosphodiesterases increased cAMP levels in the cell and prevented attachment. Further, treatment with inhibitor did not affect the growth rate of either swimming or established attached cells, indicating that its effect is limited to a critical window during the early stages of adhesion. These data suggest that cAMP signaling is required for attachment of *C. fasciculata* and that flagellar signaling domains may be reorganized during differentiation and attachment.

**IMPORTANCE:**

Trypanosomatid parasites cause significant disease burden worldwide and require insect vectors for transmission. In the insect, parasites attach to tissues, sometimes dividing as attached cells or producing motile, infectious forms. The significance and cellular mechanisms of attachment are relatively unexplored. Here, we exploit a model trypanosomatid that attaches robustly to artificial surfaces to better understand this process. This attachment recapitulates that observed *in vivo* and can be used to define the stages and morphological features of attachment as well as conditions that impact attachment efficiency. We have identified proteins that are enriched in either swimming or attached parasites, supporting a role for the cyclic AMP signaling pathway in the transition from swimming to attached. As this pathway has already been implicated in environmental sensing and developmental transitions in trypanosomatids, our data provide new insights into activities required for parasite survival in their insect hosts.

## INTRODUCTION

Organisms from the protozoan order Trypanosomatida impose a significant disease burden especially in low-income countries (1, 2). *Trypanosoma brucei*, *Trypanosoma cruzi*, and *Leishmania* spp. are the etiological agents for African trypanosomiasis, Chagas disease, and Leishmaniasis, respectively. These parasites also cause important veterinary diseases (3), while still others impact insects critical for pollination (4). A deeper understanding of the biology of these organisms can provide the basis for rational interventions to limit disease burden. Additionally, trypanosomatids are compelling models for basic research, representing early-branching eukaryotes that are divergent from more well-studied organisms such as insects, worms, and vertebrates (5).

Trypanosomatids primarily parasitize insects with only a handful of species using insects as a vector to infect other organisms (6, 7). In insects, trypanosomatids spend part of their life cycle attached to the apical surface of an epithelial lining. Attachment often precedes a developmental transition, including distinct morphological and molecular changes [reviewed in Ref. (8)] For example, in the kissing bug *Rhodnius prolixus*, *T. cruzi* parasites bind to the perimicrovillar membrane in the posterior midgut, followed by a more stable attachment to the cuticular lining of the hindgut, before being excreted by the insect as infectious metacyclics (9, 10). Similarly, *T. brucei* attaches to the salivary gland epithelium, and *Leishmania* spp. attach to the stomodeal valve (11). In each case, attachment is temporally correlated with differentiation to the final developmental form in the insect. Despite the importance of attachment in the trypanosomatid insect life cycle, the relative inaccessibility of these parasite stages in the insect means that little is known about attachment mechanisms and the signal transduction pathways that connect attachment to developmental progression.

*Crithidia fasciculata* is a monoxenous parasite of mosquitoes that developmentally transitions from a swimming (nectomonad) cell with a long flagellum to an attached (haptomonad) cell with a dramatically shortened flagellum. The tip of this flagellum contains a hemidesmosome-like attachment plaque anchoring the cell to the cuticular lining of the hindgut epithelium. A morphologically similar plaque is found in attached forms of other trypanosomatid species (8, 12). Remarkably, for *C. fasciculata,* the developmental switch from a swimming to an attached cell is readily produced *in vitro* by prolonged contact with a variety of substrates, including Millipore filters (13), culture debris (14), and plastic dishes (15, 16). Taking advantage of the ability to produce large numbers of swimming and attached cells in culture, our prior transcriptomic analysis identified genes encoding putative cyclic adenosine monophosphate (cAMP) signaling components as differentially regulated in the different forms of *C. fasciculata*. This led to the hypothesis that cAMP signaling may be involved in cell fate transitions in this organism (15).

Despite intense study and increased knowledge about the critical roles of cAMP signaling in trypanosomatids, much remains unknown (17). As in other organisms, the cAMP second messenger is created from ATP by adenylate cyclases (ACs). In trypanosomatids, many ACs are transmembrane adenylate cyclases (RACs) that may act as receptors for unidentified ligands. The RAC family of ACs is dramatically expanded in *T. brucei*, in which they localize to the flagellum, either along the length or at the distal tip (18). In *Trypanosoma*, protein kinase A (PKA) is not activated by cAMP (19). Instead, cAMP response proteins (CARPs) are thought to mediate downstream events (20–22). Phosphodiesterases (PDEs) terminate the signal by degrading cAMP to 5′-AMP. In *T. brucei*, some of these PDEs are localized to the flagellum, while others are found in the cell body (23). It has been proposed that PDEs act to limit the diffusion of cAMP and create spatially restricted signaling microdomains (24, 25). This paradigm has also been demonstrated in other eukaryotic systems (26).

Using a robust and quantitative attachment assay and imaging of live and fixed cells, we have defined the process of *C. fasciculata* attachment and the conditions that promote it *in vitro*. In addition, we have confirmed differential regulation of cAMP signaling components at the protein level and have used pharmacological inhibitors to probe the role of cAMP signaling during the transition to an attached state. Finally, we show the localization of putative components of the *C. fasciculata* cAMP signaling pathway to the flagellum in swimming cells and show striking changes in the distribution of these proteins upon flagellar shortening. These findings support the use of *C. fasciculata* as a model to understand the spatial regulation of signal transduction in the context of attachment and differentiation of kinetoplastid parasites.

## RESULTS

### Culture conditions impact *in vitro* attachment of *C. fasciculata*

**Fig 1:**
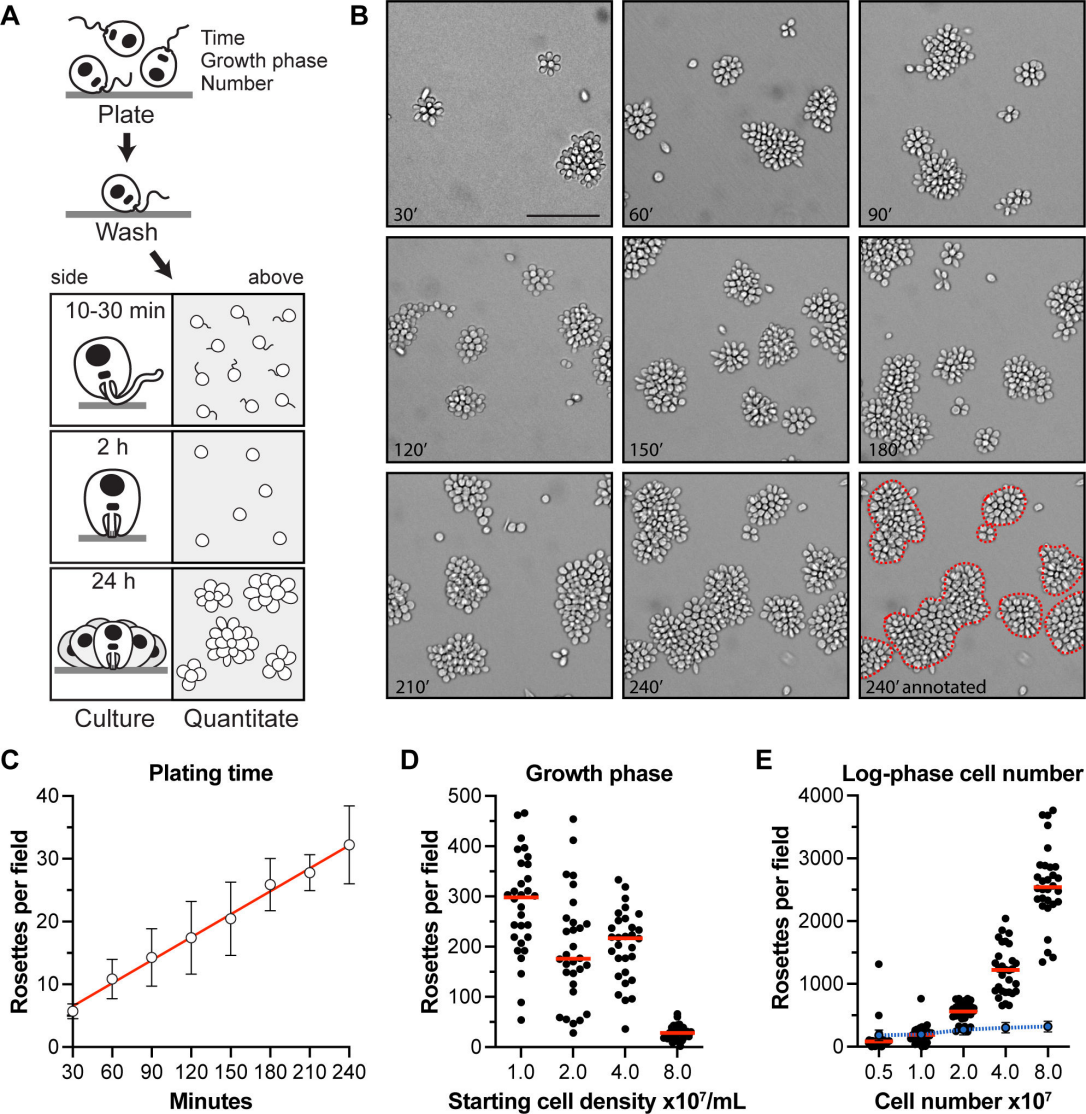
An adherence assay reveals features of *C. fasciculata* attachment. (**A**) Schematic showing steps of the assay, including the variables tested. (**B**) Representative images showing attached rosettes produced by varying the initial plating time. The dashed red lines were drawn to indicate what was counted manually as individual rosettes, defined as clusters containing four or more cells. (**C**) Quantitation of the experiment shown in (**B**). This experiment was performed in triplicate. Error bars show standard error. (**D**) The effect of initial cell concentration on adherence. 10^7^ cells/mL from cultures in different phases of growth were adhered for each sample. Quantitation of a representative experiment of three replicates is shown with median values indicated by red lines. (**E**) Rosettes resulting from adherence of different numbers of cells, all derived from a log phase culture (10^7^ cells/mL). A representative experiment of three replicates is shown. Red lines indicate median values, and the blue dotted line represents the proportion of plated cells that were able to adhere.

To probe the mechanism of *C. fasciculata* attachment, we used an *in vitro* assay to determine which conditions impact attachment rate. Swimming cells, grown on a shaker, were transferred to fresh culture plates and incubated without shaking to allow cells to contact the surface. Plates were washed to remove unattached cells, and the number of single attached cells in representative fields were quantitated (Fig. 1A). Over the next 24 h, these single cells divided to form attached rosettes. Cells in a rosette can undergo division to produce either two attached cells or two swimming cells that can leave the rosette (15). Some proportion of these newly produced swimming cells can also encounter the dish and become attached. In our standard assay, swimming cells were again washed away at 24 h. Imaging representative fields allowed us to quantitate the number of rosettes (clusters of four or more cells).

We found that the length of the initial incubation prior to washing was directly proportional to the number of attached parasites, with 240 min producing the greatest number of rosettes (Fig. 1B and C). Consistent with a previous report (27), we also found that the growth phase of the cultured cells had a strong effect on the rate of attachment. Cells from a log phase culture (10^7^ cells/mL) adhered and differentiated at a higher frequency compared with the same number of cells taken from a stationary phase culture (10^8^ cells/mL, Fig. 1D). In addition to having a reduced rate of cell division, stationary phase cells are morphologically distinct from cells in exponentially growing cultures, with the latter being rounder and shorter (Fig. S1). Increasing the total number of cells used in the attachment assay, provided those cells were taken from a log-phase culture, proportionally increased the total number of attached cells but had relatively little impact on the percentage of cells that attached during the 2-h incubation (Fig. 1E). These data suggest that *in vitro* attachment of *C. fasciculata* happens at a constant rate.

**Fig 2:**
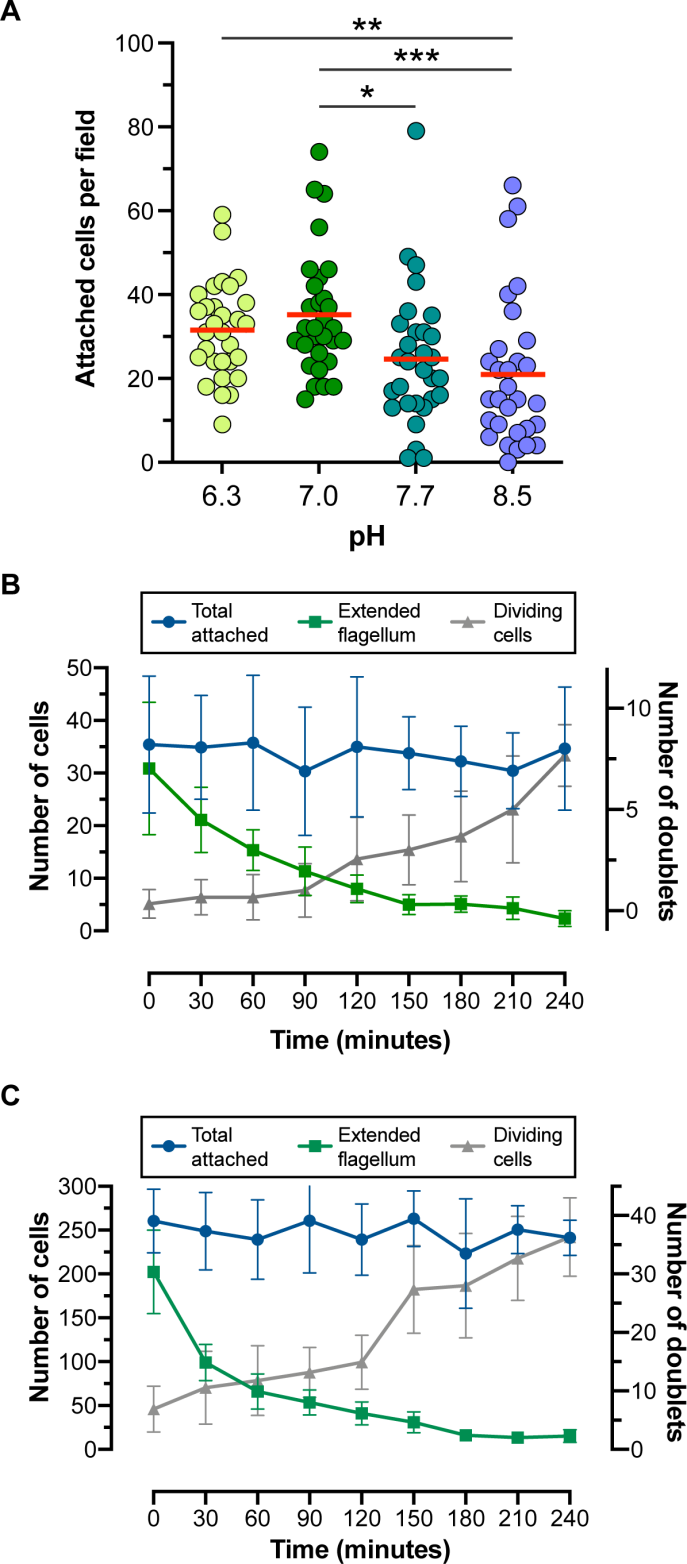
Culture conditions can impact attachment. (A) Log-phase cells were allowed to attach for 2 hours in BHI medium adjusted to the indicated pH levels. Following washing with PBS, plates were imaged for single attached cells. *P < 0.05; **P < 0.01. Kruskal–Wallis. (B) Standard attachment assay performed in non-treated flasks or (C) tissue culture-treated flasks. Log phase cells were allowed to attach for 2 hours, followed by washing and imaging for quantitation. The mean of two replicates is shown. Error bars are standard error.

To test if exposure to low or high pH impacts attachment efficiency, we performed our standard assay in media with varying pHs (Fig. 2A). There was significantly less attachment at pH 8.5 compared with pH 7, while reduction of the media pH to 6.3 had no effect. We next asked if artificial substrates with different properties affect attachment rates. We incubated cells from a log-phase culture in either untreated plates or plates that had been treated with gas plasma to increase their hydrophilicity (hereafter referred to as treated plates). After 2 h, we washed away swimming cells and imaged three random fields. We continued to image three random fields per plate in 30-min increments thereafter, counting the total number of attached cells, those that still had an extended flagellum, and attached cells that had undergone division to produce doublets (Fig. 2B and C). Across two experiments, treated plates had a larger number of attached cells (note the differences in the y-axes in Fig. 2B and C), suggesting that this surface supports efficient *in vitro* attachment. In both types of plates, we found that the number of cells with extended flagella started to decrease at the first time point (30 min after washing) although the drop was slightly steeper in the treated plates. At early time points, the number of dividing cells (doublets) was low but increased steadily. A typical developmental transition therefore involves surface contact, flagellar retraction, and finally division to produce more attached cells.

**Fig 3:**
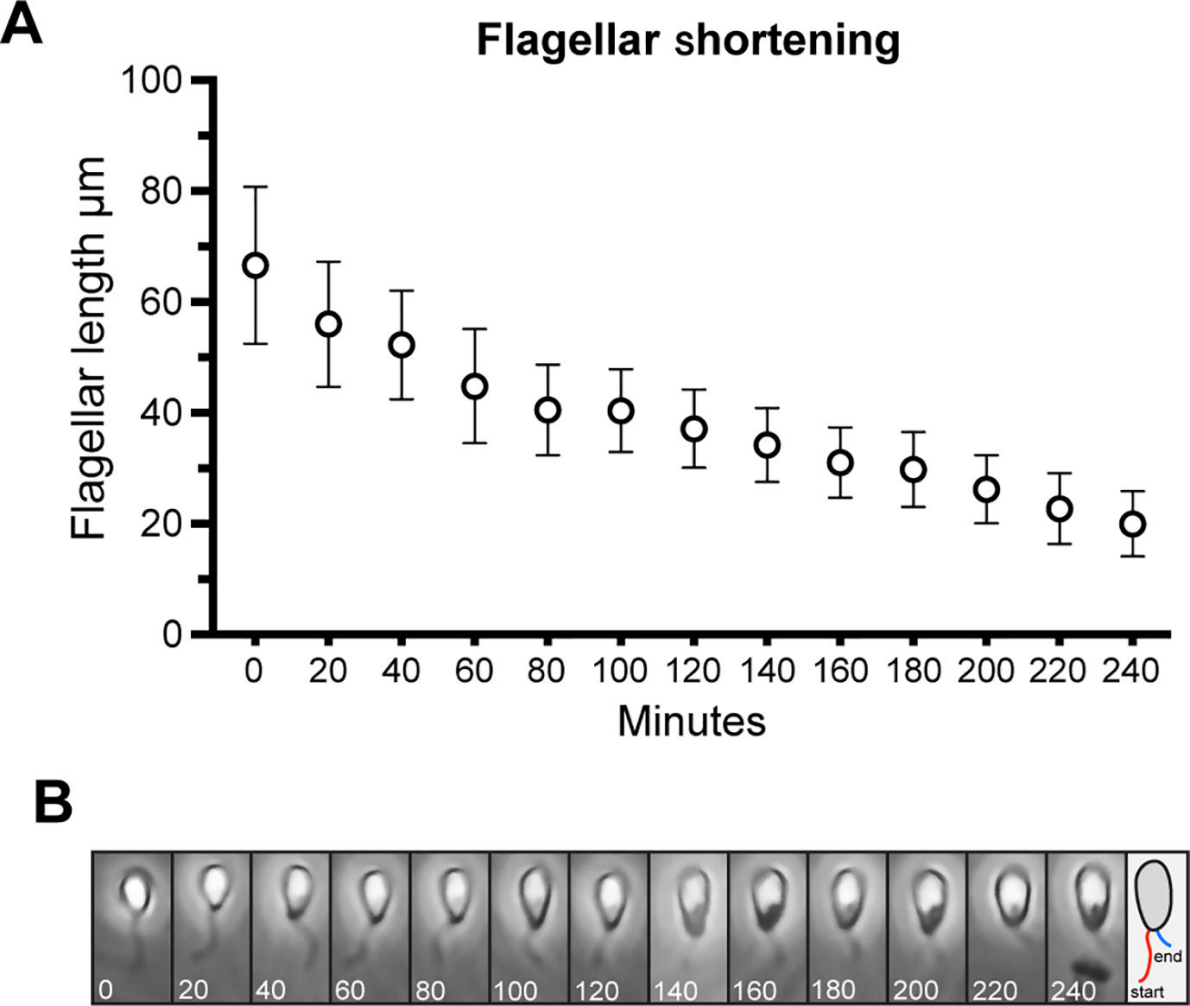
Flagellar shortening occurs at a relatively constant rate following an initial adhesion event. (**A**) Following 5 minutes of plating and subsequent washing, cells were observed at the indicated times and the length of the flagellum was measured in 20 representative cells (different cells were measured at each time point). (**B**) An example of a single cell undergoing flagellar shortening. The numbers indicate time in minutes. The schematic on the right shows a representation of the length of the flagellum at the beginning (red) and end (blue) of the time course.

To confirm this, we used time-lapse microscopy to follow parasites in environmentally controlled conditions (neutral pH, 27°C, non-treated plastic). Using flagellar shortening and cell rounding as morphological markers for differentiation, we observed that these events were always preceded by an initial event during which parasites had sustained contact with the surface *via* their anterior end, near the flagellar pocket (Videos S1 and S2). During flagellar retraction, the cells become rounder before undergoing their first cellular division as attached cells. In certain cases, cell division occurs prior to the full retraction of the flagellum, but this was rare. Parasites sometimes attach in pairs, a phenomenon that has been reported previously (28). In this case, one cell of the pair will typically detach and swim away (Video S2). From these experiments, it was possible to directly measure the rate of flagellar shortening, revealing that the flagellum retracts at a relatively constant rate until around 2 h (Fig. 3).

### Structure of *C. fasciculata* attachment *in vitro*

**Fig 4:**
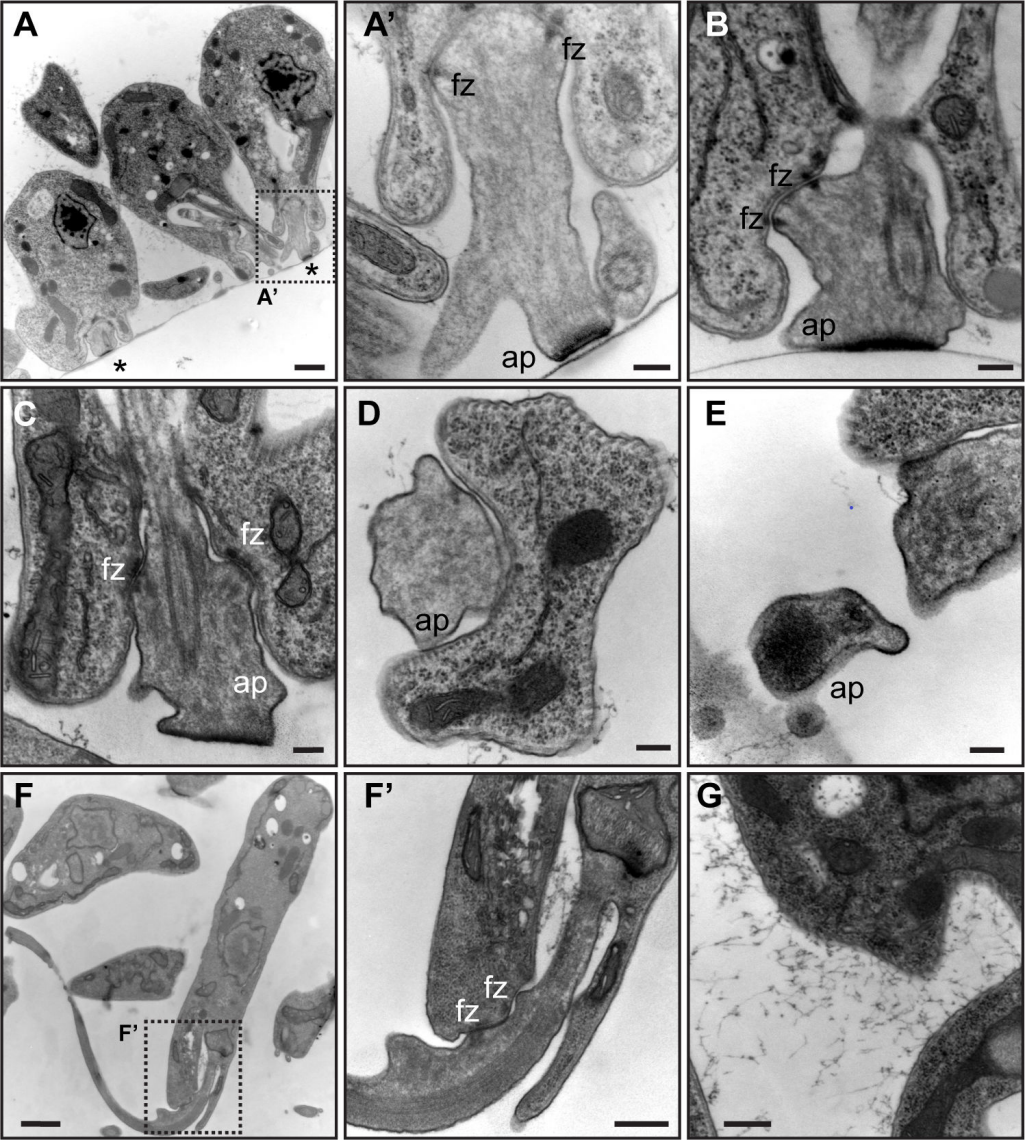
Transmission electron microscopy reveals structural differences between attached and swimming cells. (A) Cross-section through *C. fasciculata* parasites attached to tissue culture plastic (Fig. S2, yellow plane). Asterisks indicate attachment plaques at the distal tip of the shortened flagella. Scale bar is 1 µm. The dotted box indicates the region enlarged in panel (A’). (A’) Close up of an attachment plaque (ap) from one of the cells in panel A. FAZ structures, connecting the flagellum to the cell body, are also observed (fz). Scale bar is 200 nm. (B) and (C) Additional examples of attachment plaques (ap) and FAZ (fz). The cell in (C) was scraped from the dish prior to fixation. Scale bar is 200 nm. (D) Sectioning *en face* shows a region near the end of the flagellum in an adherent cell (Fig. S2, blue planes). The flagellar membrane is enlarged, and no flagellar axoneme can be observed. Instead, diffuse filaments probably comprising the attachment plaque are seen. Scale bar is 200 nm. (E) Another example of an attachment plaque sectioned *en face*. Scale bar is 200 nm. (F) Section of a swimming cell shows the flagellum extending from the flagellar pocket. Dotted box indicates the region enlarged in (F’). Scale bar is 1 µm. (F’) Higher magnification view of the flagellar pocket of a swimming cell. FAZ structures link the flagellar membrane to the flagellar pocket membrane, but no hemidesmosome is seen. The slight bulge in the flagellum where it exits the pocket may be a physical correlate to the site of initial adhesion. Filamentous material can be observed in the flagellar pocket of the swimming cell but was never observed on the surface. In contrast, the surface of attached cells (G) was covered with this material. Some of the same material is also visible in panels (A–E). Scale bar of F’ and G is 400 nm.

While parasites initially adhere near the base of their flagellum, stably attached cells are connected to the substrate *via* the distal tip of the shortened flagellum. Previous reports on *C. fasciculata* and other trypanosomatids have described plaques at the site of attachment that ultrastructurally resemble hemidesmosomes (8, 11, 14). Since most imaging of the attachment structure was performed on parasites in the insect, we used transmission electron microscopy (TEM) to determine if attachment plaques were present *in vitro*. Consistent with findings in other trypanosomatids (11, 16), a cross-section of rosettes showed an osmophilic, filamentous plaque at the distal tip of the shortened flagellum adjacent to the substrate (Fig. 4A and B). When we detached cells from culture plates by scraping prior to fixation, the hemidesmosome appeared intact, indicating that attachment mediated by this structure can be physically disrupted (Fig. 4C). Fibrous, desmosome-like structures appear to connect the proximal part of the flagellum to the cell body at the flagellar pocket, features which have also been noted in prior studies and which are part of the flagellar attachment zone (FAZ) (Fig. 4A through C) (14, 29, 30). When cells were sectioned *en face* near the attachment site with the culture plate, we observed that the distal portion of the flagellum containing the plaque was somewhat enlarged and extended beyond the termination point of flagellar axoneme (Fig. 4D). In these sections, attachment plaques are circular, and the degree to which they are stained by uranyl acetate is likely a function of how close to the culture plate they were sectioned (Fig. 4D and E). By contrast, in swimming cells, we only found FAZ-like structures connecting the flagellum to the pocket but did not observe plaques at the distal end of the flagellum (Fig. 4F). A notable feature of both attached cells and scraped attached cells was an extracellular network of filamentous material attached to the plasma membrane of the cell body (Fig. 4A through D and G). Similar material has been described for attached forms of other species (31, 32). In swimming cells, similar material was sometimes observed in the flagellar pocket itself, but not on the cell surface (Fig. 4F).

### Proteomics reveals developmentally regulated metabolic and signaling proteins

**Fig 5:**
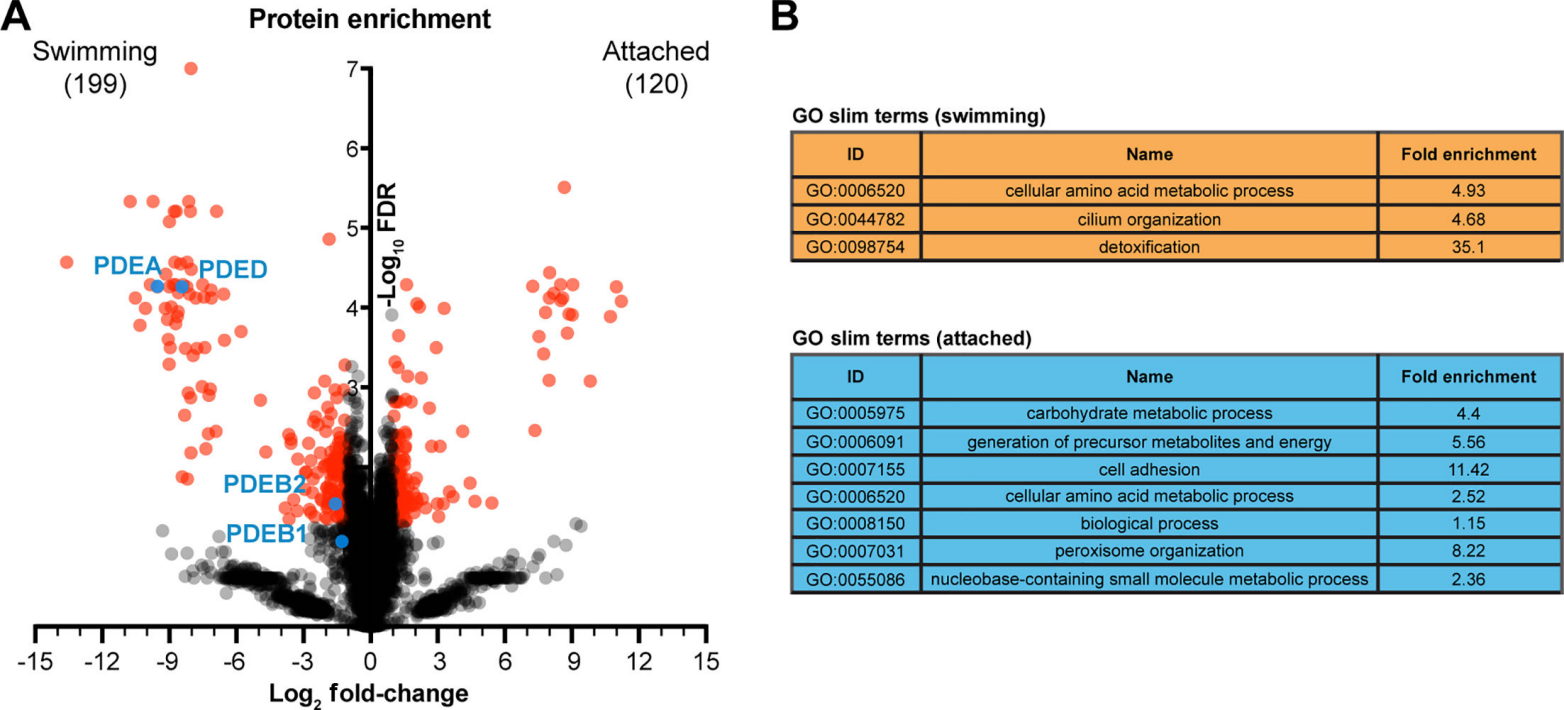
Proteomics analysis of swimming and attached *C. fasciculata*. (**A**) Volcano plot highlighting differentially abundant proteins upregulated in attached forms (right side) versus swimming forms (left side). Four cAMP phosphodiesterases are indicated. (**B**) Trimmed gene ontology (GO) terms associated with proteins upregulated in swimming (orange) or attached (blue) parasites.

We previously performed transcriptomic analysis on *C. fasciculata* to identify differentially regulated transcripts in cultured swimming, cultured attached, and parasites attached *in vivo* to their mosquito host (15). This list provided candidates, including transcripts for GP63 surface proteases (upregulated in attached cells) and cAMP signal transduction components (upregulated in swimming cells), that suggest underlying biological processes involved in attachment. To validate and prioritize these candidates, we performed semi-quantitative label-free mass spectrometry on proteins extracted from three biological replicates of either swimming or attached *C. fasciculata* grown in culture. By applying a threshold of a minimum absolute fold change of 2 and a q-value <0.05, we identified 120 distinct proteins upregulated in attached cells relative to swimming cells and ~199 proteins upregulated in swimming cells relative to attached cells (Fig. 5A; Table 1; Table S1). Some of these include multiple sequences from protein families for which mapped peptides could not distinguish between individual proteins. We analyzed these lists using gene ontology (GO) and found that many proteins upregulated in swimming cells have predicted functions in amino acid metabolism, while proteins more abundant in attached cells were predicted to mediate carbohydrate metabolism, including a number of predicted glycosomal proteins (Fig. 5B; Table 2; Table S1). Although there was limited overlap in differentially regulated transcripts detected by RNAseq and proteomics analyses, the “cell adhesion” GO category for proteins enriched in attached cells consists entirely of a group of closely related putative GP63 surface proteases. Similarly, in swimming cell protein samples, three cAMP PDEs were identified as significantly enriched (Fig. 5A).

**TABLE 1:** Top differentially expressed proteins (swimming cells)

Protein IDs	Product description	LFQ fold change attached/swimmers
CFAC1_020010300	Hypothetical protein	−12,375.36
CFAC1_210023200	Amastin-like surface protein-like protein	−1,731.15
CFAC1_230012400	Hypothetical protein, conserved	−1,473.64
CFAC1_200010400	Hypothetical protein, conserved	−1,274.05
CFAC1_300070400	Short-chain dehydrogenase, putative	−1,076.42
CFAC1_140014700	Serine/threonine kinase-like protein, putative	−923.17
CFAC1_280034100	Hypothetical protein, conserved	−849.56
CFAC1_140020300	cAMP phosphodiesterase A, putative	−738.78
CFAC1_210042200	Hypothetical protein, conserved	−738.13
CFAC1_260041100	3-Oxoacid CoA-transferase, A subunit, putative	−581.67
CFAC1_300006000	Dynein regulatory complex subunit, putative	−569.37
CFAC1_200009000	Mitochondrial-associated ribonuclease, putative	−539.94
CFAC1_210020300	Hypothetical protein, conserved	−530.22
CFAC1_200011300	Complex1_LYR-like, putative	−516.18
CFAC1_300090200	Protein kinase A catalytic subunit isoform 2	−511.25
CFAC1_290046500	d-Isomer-specific 2-hydroxyacid dehydrogenase-like protein	−510.92
CFAC1_270013600, CFAC1_270013800	Unspecified product	−496.37
CFAC1_180009800	Major facilitator superfamily, putative	−479.48
CFAC1_020023800	Repeat of unknown function (DUF1126), putative	−451.98
CFAC1_210006100	Calmodulin-binding, putative	−438.41
CFAC1_300073000	WD domain, G-beta repeat, putative	−430.88
CFAC1_210037100	LIM domain-containing protein, putative	−423.33
CFAC1_130022800	Cytochrome b5-like heme/ steroid-binding domain-containing protein, putative	−420.49
CFAC1_280050700	Elongation factor-2 kinase-like protein	−412.75
CFAC1_050021800	Hypothetical protein, conserved	−399.23
CFAC1_060010500	TLD domain protein, conserved	−388.46
CFAC1_290010800	Leucine-rich repeat, putative	−386.63
CFAC1_230026900	Hypothetical protein, conserved	−361.37
CFAC1_200032200	3'5'-Cyclic nucleotide phosphodiesterase, putative	−344.83
CFAC1_080008900	Hypothetical protein, conserved	−342.63
CFAC1_020006500	Paraflagellar rod component par4, putative	−335.39
CFAC1_290059000	Hypothetical protein, conserved	−317.94
CFAC1_300086100	Unspecified product	−311.35
CFAC1_250050300	Hypothetical protein, conserved	−297.05
CFAC1_260024500	Ciliary BBSome complex subunit 2, N-terminal/Ciliary BBSome complex subunit 2, middle region/Ciliary BBSome complex subunit 2, C-terminal, putative	−294.03
CFAC1_230038900	Retinoic acid induced 16-like protein, putative	−293.37
CFAC1_160013800	Soluble NSF attachment protein, SNAP, putative	−284.67
CFAC1_100025900	WD domain, G-beta repeat, putative	−279.73
CFAC1_300105600	Dynein heavy chain and region D6 of dynein motor/ Ankyrin repeats (three copies), putative	−273.3
CFAC1_300056400	IQ calmodulin-binding motif containing protein, putative	−266.7
CFAC1_240008100	Ring finger-containing protein, putative	−264.5
CFAC1_280014800	BRE1 E3 ubiquitin ligase, putative	−262.57
CFAC1_030006000	Folate/biopterin transporter, putative	−262.19
CFAC1_240006600	Hypothetical protein, conserved	−260.7
CFAC1_300103200	Folate/biopterin transporter, putative	−244.57
CFAC1_260039300	DNAJ domain protein, putative	−225.09
CFAC1_200032000	Acyltransferase, putative	−216.87
CFAC1_260050300	Hypothetical protein, conserved	−185.41
CFAC1_160018700	Hypothetical protein, conserved	−182.71

**TABLE 2:** Top differentially expressed proteins (attached cells)

Protein IDs	Product description	LFQ fold change attached/swimmers
CFAC1_110026300, CFAC1_110026400	Folate/biopterin transporter, putative	2,385.26
CFAC1_230008400	Hypothetical protein, conserved	2,040.3
CFAC1_240006100	Folate/biopterin transporter, putative	1,691.77
CFAC1_210045400	Fatty acid desaturase, putative	911.02
CFAC1_030012700	CLN3 protein, putative	527.68
CFAC1_100012500, CFAC1_100012600	Protein kinase, putative	521.34
CFAC1_210012900	Protein of unknown function (DUF3184), putative	468.83
CFAC1_260054800	Lsm5p, putative	444.83
CFAC1_220028200	Pumilio/PUF RNA-binding protein 5, putative	406.43
CFAC1_040011500, CFAC1_040011600, CFAC1_040011900, CFAC1_040012000	GP63, leishmanolysin	385.1
CFAC1_300100600	Tubulin tyrosine ligase, putative	365.31
CFAC1_170013900	Kinesin motor domain-containing protein, putative	359.96
CFAC1_260010500	Ubiquitin-conjugating enzyme-like protein	293.2
CFAC1_260011600	Hypothetical protein, conserved	257.06
CFAC1_250008300	Hypothetical protein, conserved	255.86
CFAC1_300045000	Beta-lactamase, putative	254.27
CFAC1_040015500	Hypothetical protein, conserved	226.43
CFAC1_220037700	Hypothetical protein, conserved	214.12
CFAC1_150031500	Hypothetical protein	185.59
CFAC1_300011500	DNA repair protein RAD51, putative	162.76
CFAC1_150024800	Hypothetical protein, conserved	152.01
CFAC1_040011700	GP63, leishmanolysin	42.93
CFAC1_300086000	Fusaric acid resistance protein-like, putative	25.41
CFAC1_250021600	Iron-containing alcohol dehydrogenase, putative	21.78
CFAC1_270037000	Amino acid permease	17.38
CFAC1_050023300	ATP-dependent helicase, putative	12.9
CFAC1_290055100	Hypothetical protein, conserved	11.51
CFAC1_210016900	Malic enzyme, putative	9.82
CFAC1_220045900	Squalene monooxygenase-like protein	9.58
CFAC1_280071100	Glucose transporter 3	8.6
CFAC1_230020800	Cysteine desulfhydrase	8.24
CFAC1_110007200, CFAC1_110008000, CFAC1_110007300, CFAC1_110007900, CFAC1_110007800, CFAC1_110007400, CFAC1_110007600, CFAC1_110007000	Carboxypeptidase, putative Carboxypeptidase Taq (M32) metallopeptidase, putative Hypothetical protein	7.92
CFAC1_050026400	Cytochrome b5-like protein (pseudogene)	7.68
CFAC1_160017800, CFAC1_160017700	Sphingolipid delta 4 desaturase, putative	6.62
CFAC1_240005900	Coproporphyrinogen III oxidase	6.22
CFAC1_230036900	Cupin-like domain containing protein, putative	5.52
CFAC1_210036700	Enoyl-CoA hydratase/enoyl-CoA isomerase/3- hydroxyacyl-CoA dehydrogenase, putative	4.97

### cAMP signaling proteins localize to the flagellum

**Fig 6:**
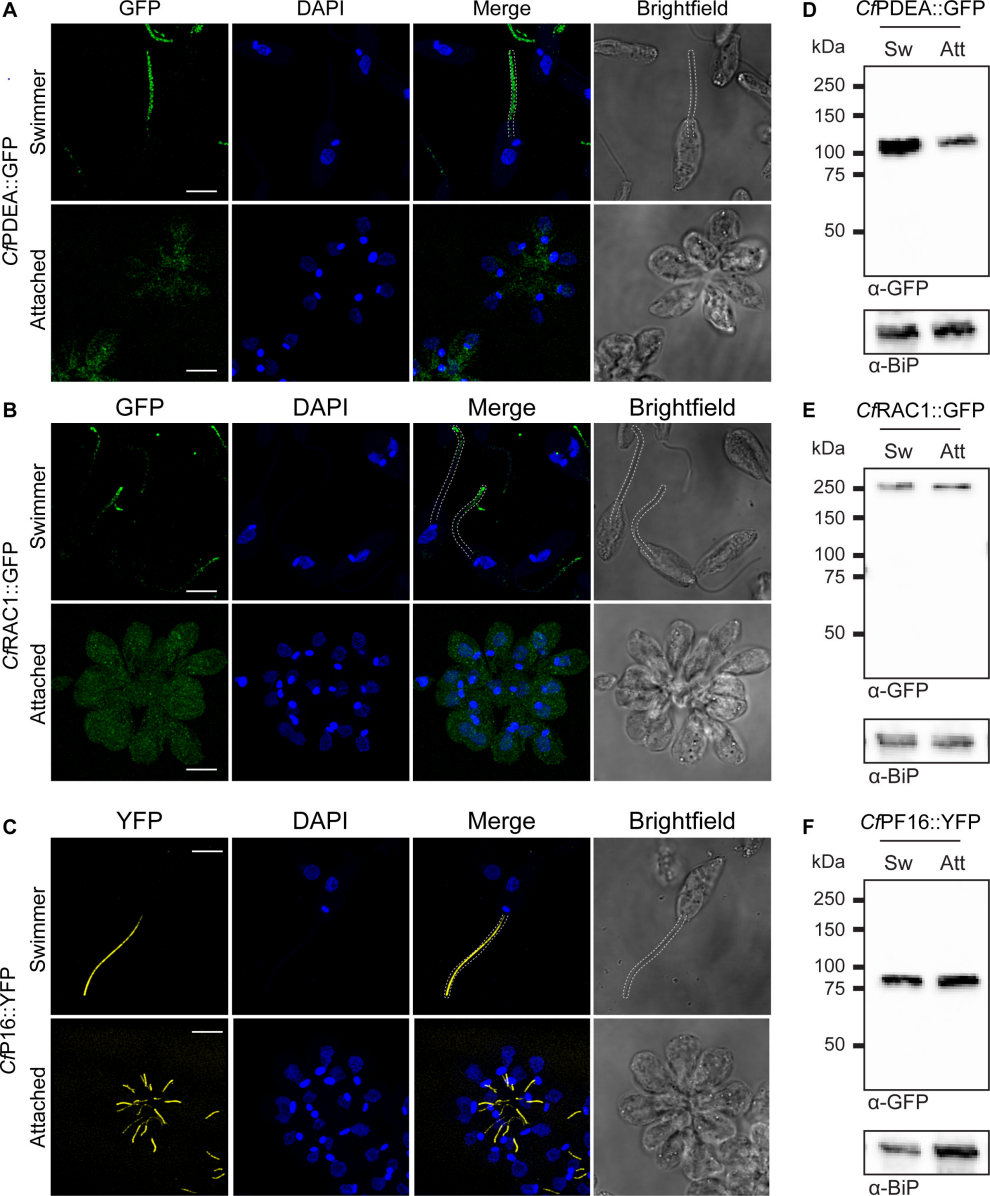
Components of cAMP signaling localize to different domains of the flagellum in *C. fasciculata* swimmers. confocal images of (A) *Cf*PDEA::GFP, (B) *Cf*RAC1::GFP, and (C) *Cf*PF16::YFP fusion proteins with DAPI staining of nuclear and kDNA of fixed swimmer and attached cell cultures. A Z-stack of the entire cell or rosette was captured. Maximum projections of individual deconvolved images are shown. A brightfield image for each field is included for reference and was used to generate the white dashed outline of the flagellum in the merged and brightfield images. Scale bar 5 µm. (D–F) Western blots of extracts of swimmers (Sw) and attached (Att) cells of each line probed with an antibody to GFP (α-GFP) or BiP (α-BiP) as a loading control.

In other trypanosomatids, components of cAMP signaling pathways are localized to flagellar subdomains, indicating that this organelle may be involved in spatially restricting the distribution of this second messenger (18, 23). To see if this feature is also present in *C. fasciculata*, we localized putative cAMP phosphodiesterase *Cf*PDEA (CFAC1_140020300), which was one of the top differentially expressed proteins found in swimming cells, by fusing a GFP protein to its C-terminus and expressing this fusion as an ectopic copy (Fig. S3). In swimming *C. fasciculata*, *Cf*PDEA::GFP is found along the length of the flagellum, often appearing as two parallel lines of puncta (Fig. 6). The extent of *Cf*PDEA::GFP is somewhat diminished along the portion of the flagellum in the flagellar pocket. We also localized a putative receptor adenylate cyclase we named *Cf*RAC1 (CFAC1_090006500). There are six predicted RACs in the draft *C. fasciculata* genome assembly, as well as three receptor adenylate cyclase a-like proteins. The six putative RACs are found in the same region of the chromosome in a tandem array. We chose the first gene in the array for tagging because it has the most diverged N-terminus and 5′ UTR. *Cf*RAC1::GFP was also found in puncta, but was restricted to the distal third of the flagellum in swimming parasites (Fig. 6; Fig. S4). We compared the localization patterns of *Cf*PDEA::GFP and *Cf*RAC1::GFP to another cell line in which a YFP sequence was fused to one of the endogenous copies of the *Cf*PF16 gene (Fig. S4), a component of the central pair of the flagellar axoneme (33). *Cf*PF16::YFP was uniformly distributed along the length of the flagellum in a single continuous line without obvious puncta. No fluorescent signal was detected in parental CfC1 cells (Fig. S5).

We then followed these localization patterns as cells transitioned from the swimming to the attached form. As expected, *Cf*PF16::YFP could still be detected in the shortened flagellum, which, as seen in our EM studies, retains the central pair. However, *Cf*PDEA::GFP was no longer localized to the flagellum and was instead distributed across the entire cell body (Fig. 6). A similar redistribution was also observed in attached cells expressing *Cf*RAC1::GFP. To confirm that the tagged proteins are still expressed in attached cells, we performed Western blotting using an anti-GFP antibody. *Cf*PDEA::GFP and *Cf*RAC1::GFP are readily detected in both swimming and attached parasites despite their different subcellular localizations (Fig. 6). No fluorescent or anti-GFP Western signal was observed in the parental cells (Fig. S6).

### Pharmacological inhibition of PDEs blocks adherence without affecting growth

**Fig 7:**
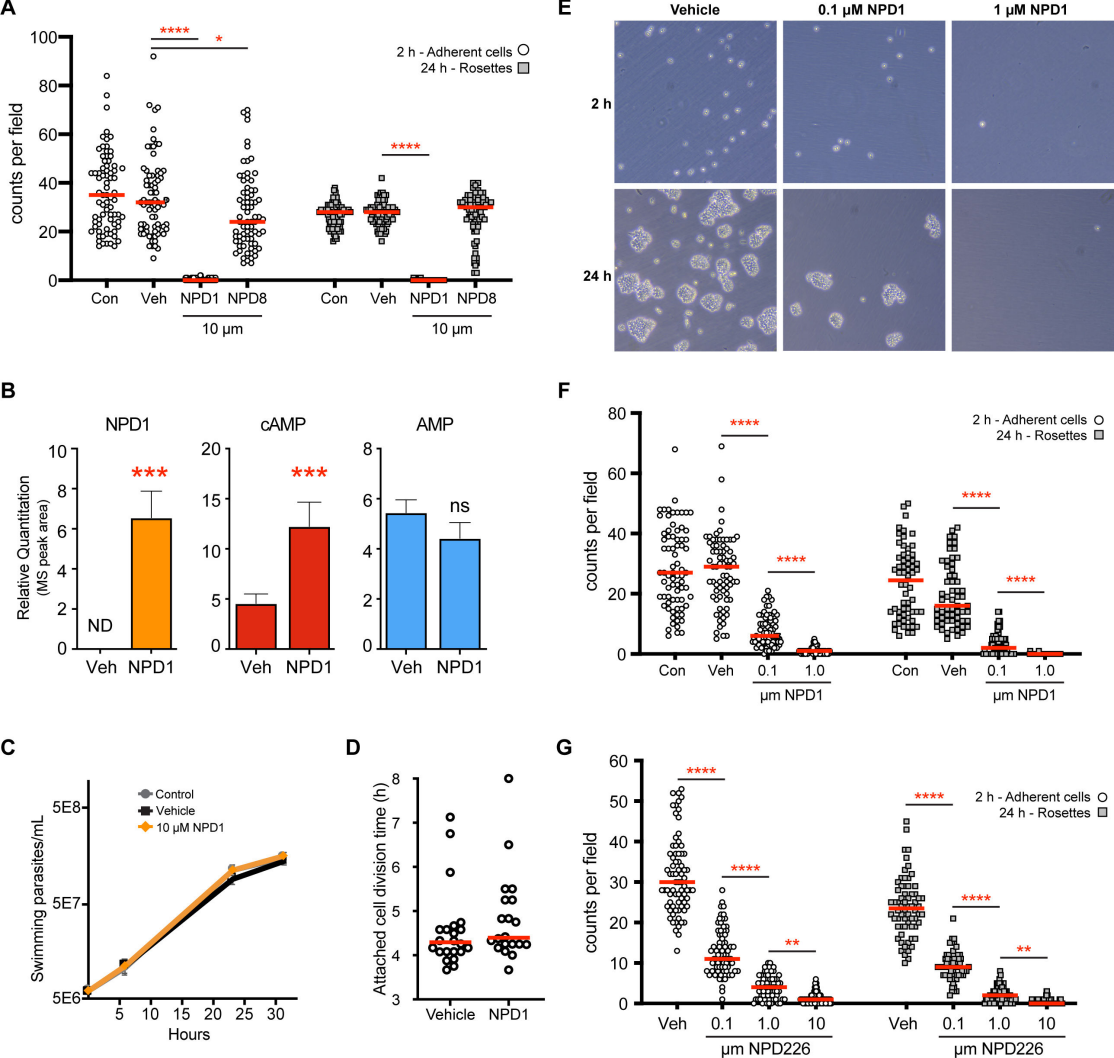
Phosphodiesterase inhibitors block *C. fasciculata* adherence. (A) Attachment assays performed in the presence of 10 µM of either NPD-001 (NPD1) or NPD-008 (NPD8). The number of single attached cells at 2 h (open circles) and the number of rosettes (≥4 cells, gray squares) at 24 h were counted and compared with those from a vehicle-treated control sample (Veh) and an untreated sample (Con). Treatments were present in the media both during the 2-h plating time and the subsequent 24 h of culture. All comparisons were made. Asterisks indicate Kruskal-– test with Dunn’s correction *P* < 0.0001 (****) and *P* < 0.05 (*). (B) Mass spectrometry-based detection of NPD-001. Individual dots show the mass spectrometry (MS) peak area for NPD-001-treated (NPD1) that was consistent across all 15 samples, and DMSO-treated vehicle control that had five samples with very low peaks and 10 samples with no detectable signal. The red line indicates the mean. Peaks for cAMP (red bars) and AMP (blue bars) were converted to a fold-change between DMSO and NPD1 treatments. There was an average 3.6-fold increase in cAMP and no difference in AMP. Metabolomics with performed with three biological replicates each with five technical replicates. Error bars indicate the standard error. (C) Graph of *C. fasciculata* growth in shaking cultures assayed over approximately 31 h in standard medium (Control; gray circles), medium supplemented containing 10 µM NPD-001 in vehicle (NPD1; orange diamonds), or medium containing vehicle only (Vehicle, black squares). Mean of three replicates. Error bars show standard deviation. (D) Time-encoded movies were used to measure the doubling time of established attached cultures after the addition of NPD-001 in vehicle or in vehicle alone. The red line indicates the mean. (E) Representative images and (F) quantitation of attachment assays comparing vehicle and untreated controls to assays performed in the presence of different concentrations of NPD-001 or (G) NPD-226. The number of single adhered cells at 2 h (open circles) and the number of rosettes (≥4 cells, gray squares) at 24 h were counted. All comparisons were made, but only the groups that are significantly different from the adjacent treatment are shown. Asterisks indicate Kruskal–Wallis test with Dunn’s correction *P* < 0.0001 (****), and *P* < 0.01 (**).

To test whether manipulating the cAMP pathway could influence the ability of *C. fasciculata* to attach to plastic *in vitro,* we used previously validated compounds that are known to inhibit cAMP PDEs in the related parasite, *T. brucei* (34). As PDEs terminate the signal by degrading the cAMP second messenger, inhibition of these enzymes has been shown to increase the amount of cAMP in the parasite (35). The first compound we tested is known as NPD-001 or Compound A (CpdA) (hereafter NPD-001). The addition of 10 µM of NPD-001 to the adherence assay completely blocked parasite attachment to the plate, resulting in no single attached cells after 2 h of incubation and no visible rosettes after 24 h of growth (Fig. 7). Interestingly, a related compound, NPD-008, had only a minor effect on attachment, slightly reducing the number of single attached cells after 2 h of incubation (Fig. 7A). To confirm that NPD-001 was inhibiting phosphodiesterase activity in *C. fasciculata*, we used mass spectrometry to measure the uptake of NPD-001, the amount of cAMP, and the amount of AMP in treated versus non-treated cells (Fig. 7B). NPD-001 is detectable in the cells, and cAMP is increased ~2.5-fold; however, there was no observable change in AMP. Despite the dramatic effect on attachment, NPD-001 does not appear to be toxic to *C. fasciculata*, as swimming cells maintained on a shaker grew at a normal rate at 10 µM, the same concentration that prevents attachment (Fig. 7C). Furthermore, addition of NPD-001 to cells that were already differentiated to the attached state did not affect their attachment or their doubling time over the course of 16 h (Fig. 7D). To determine if the effect of NPD-001 on attachment was dose-dependent, we performed an attachment assay with both 1 and 0.1 µM of the compound. Although the effect of 1 µM was comparable to that of 10 µM, 0.1 µM had a moderate effect on attachment, suggesting a dose response (Fig. 7E and F). The same concentrations of a related compound, NPD-226, also significantly reduced the number of attached cells in a dose-dependent manner at both 2 and 24 h compared with vehicle-treated controls (Fig. 7G). A fourth compound, NPD-055, had a minor effect on attachment at the highest concentration (10 µM, Fig. S7).

## DISCUSSION

Attachment is a conserved stage in the trypanosomatid life cycle, suggesting a critical role (8). Most likely, trypanosomatids attach to facilitate colonization of tissues, prevent premature elimination of parasites through defecation, or allow acquisition of characteristics necessary for transmission to their next host. A mechanistic link between attachment and differentiation to infectious forms has been demonstrated in *T. cruzi* (36), but for most species, determination of attachment’s precise function awaits direct testing. The structural conservation of the flagellar attachment plaque, along with similarities in the stages of attachment, the morphology of attached cells, and the insect tissues where attached parasites are found, points to a shared evolutionary origin for this process that is deployed with variations in different species. *Leishmania* and *T. cruzi* can also use their flagellum to transiently adhere to the lining of the midgut (37, 38), an interaction that is mediated by molecules on the parasite surface (39, 40) and lectins on the surface of midgut cells (41). Ablation or blockage of these binding partners prevents transmission; however, this mode of adhesion does not involve formation of an attachment plaque and therefore is distinct from the stable attachment we describe here.

Attachment of *C. fasciculata* and other trypanosomatids to substrates *in vitro* has been noted previously (11, 14, 16, 27, 42–47). These systems provide opportunities for detailed, quantitative, and time-resolved studies of trypanosomatid attachment. *C. fasciculata* parasites initially contact the dish *via* the base of the flagellum near where it exits the cell body. We as well as others have noted a distinct bulge in this area suggesting a structurally distinct region for initial adhesion (11, 14, 46). This event is rapidly followed by flagellar shortening, cell rounding, and formation of the attachment plaque. *L. mexicana* undergoes a similar process except that adhesion sometimes occurs laterally at a position along the length of the flagellum (11). In all cases, the final differentiated form is stably attached to the substrate by a filamentous plaque that forms at the tip of the flagellum within an expanded portion of flagellar membrane (11, 42, 46, 48). As in other studies, we have found that the hemidesmosome is extremely resistant to detergents and enzymatic digestion (46) but can be physically disrupted by scraping (49). A recent study in *L. mexicana* identified three proteins, named KIAP1-3, associated with the attachment plaque itself (50). Our proteomics data did not detect differential regulation of the KIAPs, and the syntenic *C. fasciculata* ortholog of KIAP1 was enriched in swimming rather than attached parasites in our RNAseq data. As the KIAPs were identified in the attachment plaque itself following salt and detergent extraction of the cells, it is possible that these proteins do not change in abundance between swimming and attached forms, and that their function is regulated by post-translational modification and/or subcellular localization (50).

Proteomics confirmed differential regulation of cAMP phosphodiesterases, which are upregulated in swimming cells. Protein samples from swimming *C. fasciculata* were also enriched for two catalytic and one regulatory subunit of protein kinase A (PKA). However, PKA has been shown to be cAMP independent in trypanosomatids (20, 51, 52). Instead, the RNAi library screens aimed at finding novel cAMP effectors have uncovered a family of cyclic AMP response proteins (CARPs) (20, 21). Of these, the *C. fasciculata* orthologs of CARP9 (CFAC1_040020600) and CARP4 (CFAC1_22024400) are upregulated in protein samples derived from swimming parasites as are three other CARP4-like proteins with a similar domain architecture (CFAC1_230042700, CAFC1_020023800, and CFAC1_280072700).

cAMP signaling has been implicated in numerous trypanosomatid processes, including differentiation, immune evasion, social motility (SoMo), and migration in the insect [reviewed in Refs. (17, 53–55)]. Components of this signaling pathway are typically localized to the flagellum in distinct subdomains, indicating that the spatial restriction of cAMP diffusion may be a conserved feature of trypanosomatids (18, 23). In our study, both *Cf*PDEA::GFP and *Cf*RAC1::GFP display punctate localization patterns compared with *Cf*PF16::YFP. While *Cf*RAC1 has a predicted transmembrane domain, *Cf*PDEA does not. Analysis of the flagellar membrane suggests heterogeneity, which could lead to uneven localization of our tagged proteins through anchoring to lipid rafts or structures within the flagellum (56). The parallel lines of signal found in both tagged cell lines is reminiscent of intraflagellar transport trains, which associate with two sets of microtubule doublets on either side of the axoneme (57). In *T. brucei*, the phosphodiesterase *Tb*PDEB1 is an integral component of the flagellum that is resistant to detergent extraction (25), while the cAMP response protein *Tb*CARP3 is maintained at the distal tip of the flagellum by FLAgellar membrane protein 8 (FLAM8). This localization is maintained in all developmental stages in the fly except during and immediately after attachment to the tsetse salivary gland, providing another example of dynamic relocalization of cAMP signaling proteins in different developmental forms (58). It will be interesting to discover how flagellar shortening impacts cAMP signaling microdomains and how these changes might trigger or reinforce differentiated states.

In *T. cruzi*, attachment efficiency and subsequent differentiation to metacyclics are increased by nutrient deprivation (59) which, along with hyperosmotic stress, reflect conditions in the hindgut (38). Although precisely how these conditions drive attachment is unclear, cAMP signaling has been implicated in both metacyclogenesis and osmoregulation [(60–65); reviewed in Ref. (66)]. Intriguingly, a *T. cruzi* AC and a CARP have a dual localization at both the flagellar tip and in the contractile vacuole, perhaps linking environmental and osmotic sensing in this organism (67).

Strikingly, we found that treatment with CpdA/NPD-001 increases cellular cAMP levels and blocks attachment. It is important to note that NPD-001 did not increase cellular AMP levels, as products of cAMP hydrolysis including AMP were shown to trigger differentiation of *T. brucei* cells treated with cAMP analogs (68). As each of the PDE inhibitors used in our study probably acts by the same mechanism, competitively blocking the substrate-binding pocket of all parasite PDEs (69), we are unable to conclude anything from their varying effectiveness in blocking attachment. Possibly this variability can be explained by different levels of uptake of the compounds. Since treatment of stably attached *C. fasciculata* parasites with NPD-001 did not cause the cells to detach nor did it prevent division of attached cells, we hypothesize that a transient, PDE-mediated drop in cAMP is required for transition from initial adhesion to stably attached. An alternative interpretation is that elevated cAMP impacts the swimming cell population by dramatically reducing the subset of cells that are competent to adhere. A recent study on *Trypanosoma congolense*, for which attachment of bloodstream form parasites *in vitro* is thought to mimic attachment to the mammalian vasculature *in vivo*, also found that NPD-001 inhibits attachment (45). cAMP has also been shown to affect flagellar waveform in *Leishmania* (70), and NPD-001-treated *T. congolense* cells had increased motility compared with control parasites. The NPD-001-induced detachment phenotype demonstrated by *T. congolense* was phenocopied by knockdown of a putative mRNA-binding protein and differentiation regulator, *Tcon*REG9.1, suggesting it may act downstream of cAMP signaling events to mediate changes in RNA abundance required for differentiation.

In summary, we have provided evidence that cAMP signaling regulates attachment of *C. fasciculata*, a function that may be conserved among trypanosomatids. Clearly, the spatiotemporal regulation of this key signaling pathway contributes to multiple critical processes, including environmental sensing, motility, attachment, and differentiation. Future parallel studies in multiple species will hopefully help us to disentangle the mechanisms underlying integration and transduction of signaling events and how these impact shared aspects of trypanosomatid biology and well as the diverse adaptations of different species.

## MATERIALS AND METHODS

### *Crithidia* maintenance

The Cf–C1 strain of *C. fasciculata* was used for all experiments in this study. This strain was originally obtained from Dr. Stephen Beverley and was grown in complete medium consisting of brain heart infusion (BHI) medium (Sigma) supplemented with 20 µg/mL hemin (Sigma) and penicillin/streptomycin (Millipore Sigma P4458 diluted 100×). Cells were maintained at 28°C in non-vented culture flasks on a rocker and were maintained at a density between 10^5^ and 10^8^ cells/mL. Swimming cells were counted on a hemocytometer following fixation in 0.3% formalin.

### Plasmids and cell lines

The construct for C-terminal YFP tagging of the *C. fasciculata* putative axoneme central apparatus protein CFAC1_170028000, named *Cf*PF16 for its shared homology to *L. mexicana* PF16 (33)*,* was created using a fusion PCR approach described previously (71). The YFP and neomycin resistance genes were amplified from pLENTv2eYFPNeo by PCR with forward primer pLENTeYFP_f and reverse primer pLENTNeo_r (Table S2). The 500 bp of homology to the C-terminus of *Cf*PF16 was amplified from *C. fasciculata* genomic DNA using forward primer PF16cterm500_f and reverse primer PF16cterm_r. 500 bp of homology to the 3′ UTR of *Cf*PF16 was amplified using PF16utr_f and PF16utr500_r. All three fragments were purified by PCR clean-up or gel extraction, and 20 ng of each fragment was combined in a fusion PCR reaction using PF16_500bpnest_f and PF16_500bpnest_r. The resulting construct, PF16eYFPNeo, was gel extracted and ethanol precipitated. To create a *C. fasciculata* CfPF16::YFP cell line, transfection was performed with 3.5 µg of PF16eYFPNeo introduced into 10^8^ cells using a Human T-cell kit and IIb Nucleofector set to program X-001 (Lonza). Other transfections described in this work used the same conditions except with *Tb*-BSF buffer (90 mM sodium phosphate, 5 mM potassium chloride, 0.15 mM calcium chloride, 50 mM HEPES, pH 7.3) (72). Immediately following transfection, cells were transferred to 30 mL of BHI without selecting drug and diluted 10-fold. For each dilution, 1 mL was plated into wells of a 24-well plate and cultured overnight without shaking. After approximately 18 h of recovery, 1 mL of BHI containing neomycin (G418) was added to each well, for a final selecting drug concentration of 25 µg/mL. Resistant clones came up in 7–14 days and were fixed and screened for eYFP fluorescence using wide-field microscopy. The resulting clonal cell line CfPF16YFP_A6 was used for these studies and was validated by linking PCR across the gene with primer PF16ORF_f binding inside the gene and reverse primer PF16UTR_r binding in the 3′UTR (outside of the area used for integration). To create the *C. fasciculata Cf*RAC1::GFP and *Cf*PDEA::GFP cell lines, the entire open reading frame of *Cf*RAC1 (CFAC1_090006500) or *Cf*PDEA (CFAC1_140020300) were amplified from *C. fasciculata* genomic DNA and cloned into the pNUSGFPcH vector (73) using NdeI and KpnI or NdeI and BglII restriction enzyme sites, respectively. Following confirmation by sequencing (Eurofins Genomics), 10 µg of the final plasmid was introduced into *C. fasciculata* as described above. Transfected cells were selected using BHI containing 80 µg/mL hygromycin. After 7 days, selecting drug concentration was increased to 200 µg/mL and cells were screened by fluorescence microscopy.

### Attachment assay

Establishment of cultures of swimming and adherent *C. fasciculata in vitro* were adapted from our previous study (15). Briefly, cells from a rocking culture of swimmers were added to 60 mm non-treated tissue culture plates (VWR) in 2 mL of BHI. For some experiments, cells were spun down in tubes at 800 rcf for 5 min and resuspended in BHI. The cell suspension was allowed to sit in the 60-mm plates undisturbed at 28°C for the indicated amounts of time during which initial adherence takes place (2 h for the standard assay). Following this incubation, each plate was washed five times with PBS before addition of fresh BHI media. When assays were performed in 24-well plates, the same protocol was followed, but the wash volume was 2 mL. Other assay parameters were tested systematically. pH was varied by modifying standard BHI media with either HCl or NAOH until the correct value was reached. To test whether tissue culture treated plastic impacted attachment, plates treated with oxygen plasma were purchased from VWR (Cat No. 10062–890), matching in every way the non-treated dishes apart from the treatment. Quantitation of adherence was performed through imaging on a Zeiss Primovert Digital Microscope. Following wash steps, plates were imaged by taking either 25 frames at 40× magnification or 10–25 frames at 20× magnification. Freshly adhered cells (singlets) were counted either manually or, when large numbers of attached cells were present, by Fiji script (33). Rosettes were counted manually as clusters of four or more cells at 24 h.

### LC MS/MS and data analysis

Sample analysis was performed at The Wistar Institute Proteomics and Metabolomics Shared Resource on a Thermo Q-Exactive HF-X mass spectrometer purchased with NIH grant S10 OD023586. Extracts for proteomics were made from log-phase swimming cells by washing 1.3 × 10^7^ cells twice with PBS and then extracting with 500 µL of non-reducing SDS-PAGE sample buffer (Pierce). Attached cell samples were generated by performing an attachment assay in a 60 mm petri dish and washing away non-attached swimmer cells after 24 h of growth on the incubator shelf followed by extraction. Triplicate samples were run 1 cm into a 10% non-reducing SDS-PAGE gel, stained with Imperial Stain (Pierce), and processed for label-free quantitative (LFQ) mass spectrometry. The entire protein-containing gel regions were excised, reduced with TCEP, alkylated with iodoacetamide, and digested in-gel with trypsin. Tryptic digests were analyzed using a 120-min LC gradient on a Thermo Q Exactive Plus mass spectrometer. Mass spectrometry data were searched with full tryptic specificity against the TriTrypDB-46 version of the *C. fasciculata* proteome database using MaxQuant version 1.6.8.0. The intensity values were adjusted by normalizing against the number of theoretical peptides for each protein. Intensity values were also normalized using LFQ intensity. For comparing protein levels between samples, the LFQ intensities were used. LFQ intensities were normalized using the MaxLFQ algorithm (PMID: 24942700). LFQ intensities were subsequently processed as Log2 transformations of the LFQ values and imputing missing values with a small value. Log2 ratio, fold-change and *t*-test p-value were calculated using the Log2 imputed LFQ intensity. A q-value was calculated by adjusting the *t*-test *P*-value to account for multiple testing using Benjamini–Hochberg FDR. Proteins were considered differentially abundant with a fold-change >2 and q-value <0.05. For metabolomics, five technical replicates were analyzed for DMSO-treated and NPD-001-treated cells, and the experiment was repeated thrice. All samples were prepared with extraction solution (80% methanol with heavy internal standards) at 20 million cells/mL. Samples were subjected to liquid chromatography (LC) using a Thermo Scientific Vanquish Horizon UHPLC system, a SeQuant ZIC-pHILIC column, a flow rate of 0.2 mL/min, and using a gradient of 20 mM ammonium carbonate, 0.1% ammonium hydroxide, pH 9.2, 5 µM medronic acid (solvent A) and acetonitrile (solvent B). The LC gradient was performed over a 26-min run time, 85% B for 2 min, 85% B to 20% B over 15 min, 20% B to 85% B over 0.1 min, 85% B for 8.9 min. For each sample, 4 µL was analyzed. Peaks were integrated using TraceFinder 4.1 with 5 ppm mass error. The mass spectrometry proteomics data have been deposited to the ProteomeXchange Consortium (74) *via* the PRIDE (75) partner repository with the data set identifier PXD055056. Gene lists were generated, and GO analysis was performed on TriTrypDB (76).

### Transmission electron microscopy and Giemsa staining

Swimming and attached *C. fasciculata* cells were processed and imaged using transmission electron microscopy (TEM) performed in the Electron Microscopy Resource Lab (Perelman School of Medicine, University of Pennsylvania). For swimming cells, 10^8^ log-phase cells were concentrated by centrifugation and resuspended in TEM fix (0.1 M sodium cacodylate buffer, pH 7.4, containing 2.5% glutaraldehyde, and 2.0% paraformaldehyde). For attached cells, rosette cultures in 60-mm dishes were established for 24 h as described above. Subsequently, 5 mL of TEM fix was added to the plate at room temperature. A second 24 h attached sample was generated by scraping cells off the plate with a disposable plastic scraper, concentrating by centrifugation, and placing in TEM fix. All samples were fixed initially at room temperature for 2 h and then placed at 4°C overnight. After subsequent buffer washes, samples were post-fixed in 2.0% osmium tetroxide with 1.5% K_3_Fe(CN)_6_ for 1 h at room temperature and rinsed in water prior to *en bloc* staining with 2% uranyl acetate. After dehydration through a graded ethanol series, the tissue was infiltrated and embedded in Embed-812 (Electron Microscopy Sciences). Thin sections were stained with uranyl acetate and SATO lead and examined with a JEOL 1010 electron microscope fitted with a Hamamatsu digital camera and AMT Advantage NanoSprint500 software. Images of cells cut in cross-section and *en face* were captured (Fig. S2). Morphological changes between log phase and stationary phase cells were obtained by performing a cytologic cytocentrifuge preparation and a standard Wright–Giemsa stain and imaging at 100×.

### High content imaging

High-content imaging was performed using an ImageXpress Micro 4-High Content imaging device (Molecular Devices) at 40× and 60× using standard 24 (CellTreat) and black welled (Vision 4tituder) 24-well plates, respectively. To measure rosette growth, cells were allowed to adhere to standard plates for 2 h followed by 5 washes with 2 mL of PBS and resuspension in fresh BHI media. Adhesion videos were obtained using black welled plates by adding 4 × 10^5^ cells to 500 µL of BHI media and allowing adhesion for 2 h prior to imaging at 1.25min intervals.

### Confocal microscopy and image processing

For fluorescence and confocal microscopy, 5 × 10^6^ swimming cells from log cultures were concentrated by centrifugation at 800 rcf for 5 min, washed once in PBS, then resuspended in PBS at a final concentration of 10^7^ cells/mL. Subsequently, 500 µL of this suspension was then pipetted onto coverslips pretreated with 0.01% poly-L-lysine. After allowing cells to attach for 20 min at room temperature, coverslips were washed twice with PBS, followed by fixation in 4% paraformaldehyde for 15 min. Coverslips were then washed again followed by permeabilization with 0.1% Triton X-100 in PBS for 5 min. Coverslips were washed in PBS prior to staining with 0.2 µg/mL DAPI in PBS for 5 min. After a final PBS wash, coverslips were mounted on a glass slide using Vectashield mounting medium (Vector Laboratories). For fluorescence microscopy of attached cells, a standard adherence assay was conducted, as described above, on 1.5 thickness glass-bottom dishes pre-coated with poly-L-lysine (MatTek). After 24 h of adherent growth, the final BHI wash contained 16% formaldehyde added to a final concentration of 4%. The plate was processed from this point as described above for the swimmer cell slides. The slides and plates were analyzed by confocal microscopy using a Leica SP5-II with a 100× objective. A Z-series was acquired for GFP/YFP, DAPI and brightfield. Following image acquisition, deconvolution analysis was performed on the fluorescent images using Huygens Essential deconvolution software (version 17.04.1p2 64b; SVI). Brightfield images are single confocal sections with a flatfield correction applied. All images of parental, *Cf*PDEA::GFP, and *Cf*RAC1::GFP were acquired with the same microscope settings. Equivalent linear adjustments were made to the brightness of each image using Photoshop Version 23.5.1 (Adobe).

### Western blot analysis

For swimming cultures, samples were generated from 2 × 10^6^ log-phase cells. For adhered cultures, an equivalent number of cells were grown on 60-mm dishes in stationary culture for 24 h and then dislodged using a cell scraper and counted by hemacytometer. Both swimming and dislodged adherent cells were transferred to microcentrifuge tubes and collected by centrifugation, washed once with PBS, then resuspended in Laemmli SDS-PAGE sample buffer at a concentration of 1 × 10^5^ cell equivalents/µL. Samples boiled for 5 min at 95°C and centrifuged briefly. Cell equivalents of ~1.5×10^6^ cells per lane were separated by 10% SDS-PAGE along with the Precision Plus Protein Kaleidoscope standard (BioRad). The fractionated proteins were transferred to a PVDF membrane. The membrane was then incubated in blocking buffer (PBS containing 5% milk and 0.2% Tween-20) for 1 h at room temperature. A mouse anti-GFP antibody (Roche) was used as the primary antibody at a 1:500 dilution in blocking buffer overnight at 4°C. Horseradish peroxidase-conjugated goat anti-mouse IgG in blocking buffer was used as the secondary antibody (1:5000). After washing, enhanced chemiluminescent substrate (BioRad) was used to develop the blots. For the loading control, the blot was reprobed with a rabbit anti-*Tb*BiP antibody (a gift of Jay Bangs) at a 1:5,000 dilution in blocking buffer, followed by washing and probing with an anti-rabbit-HRP secondary antibody (1:5,000). Images were taken by an Azure biosystems c600 using chemiluminescence settings.

### Pharmacological inhibition

Pharmacological inhibitors were dissolved in DMSO. For vehicle controls, an amount of DMSO equal to that of the most concentrated sample was added. The compounds were described previously: NPD-001 (35), NPD-008 (69), NPD-055 (77), and NPD-226 (78). For attachment assays, inhibitors were included both during the initial adhesion (2 h) and during subsequent rosette growth.

### Statistical analyses and data visualization

Prism Version 9.4.1.458 (GraphPad Software) was used to generate all plots and for all statistical tests. Plots were assembled into figures using Illustrator Version 26.5 (Adobe).

## SUPPLEMENTAL MATERIAL

Supplemental material msphere.00617-24-s0001.docx Figures S1 to S6 and legends for supplemental tables and movies.

Table S1 msphere.00617-24-s0002.xlsx Proteomics analysis showing differentially expressed proteins in swimming and attached *Crithidia fasciculata*.

Table S2 msphere.00617-24-s0003.pdf Sequences of oligonucleotide primers used to create localization constructs.

Movie S1 msphere.00617-24-s0004.mp4 Full imaging frame of *Crithidia fasciculata* parasites attaching *in vitro*.

Movie S2 msphere.00617-24-s0005.mp4 Enlarged and cropped region of the field shown in Movie S1.

ASM does not own the copyrights to Supplemental Material that may be linked to, or accessed through, an article. The authors have granted ASM a non-exclusive, world-wide license to publish the Supplemental Material files. Please contact the corresponding author directly for reuse.
