# Supplementary material for: Adhesion of Crithidia fasciculata promotes a rapid change in developmental fate driven by cAMP signaling
Source: mSphere. 2024 Sep 24;9(10):e00617-24. doi: 10.1128/msphere.00617-24 (PMC11520290; doi:10.1128/msphere.00617-24)
Supplement: Supplemental material — Figures S1 to S6 and legends for supplemental tables and movies. [file msphere.00617-24-s0001.docx]

**Figure S1.** Density dependent changes to *C. fasciculata* morphology. **A)** Giemsa-stained samples of cells at log-phase (10^7^ cells/mL) or past log-phase growth (10^8^ cells/mL). Scale bar is 10 µm. **B)** Plot of individual measured cell lengths in micrometers. Cells were measured along the**
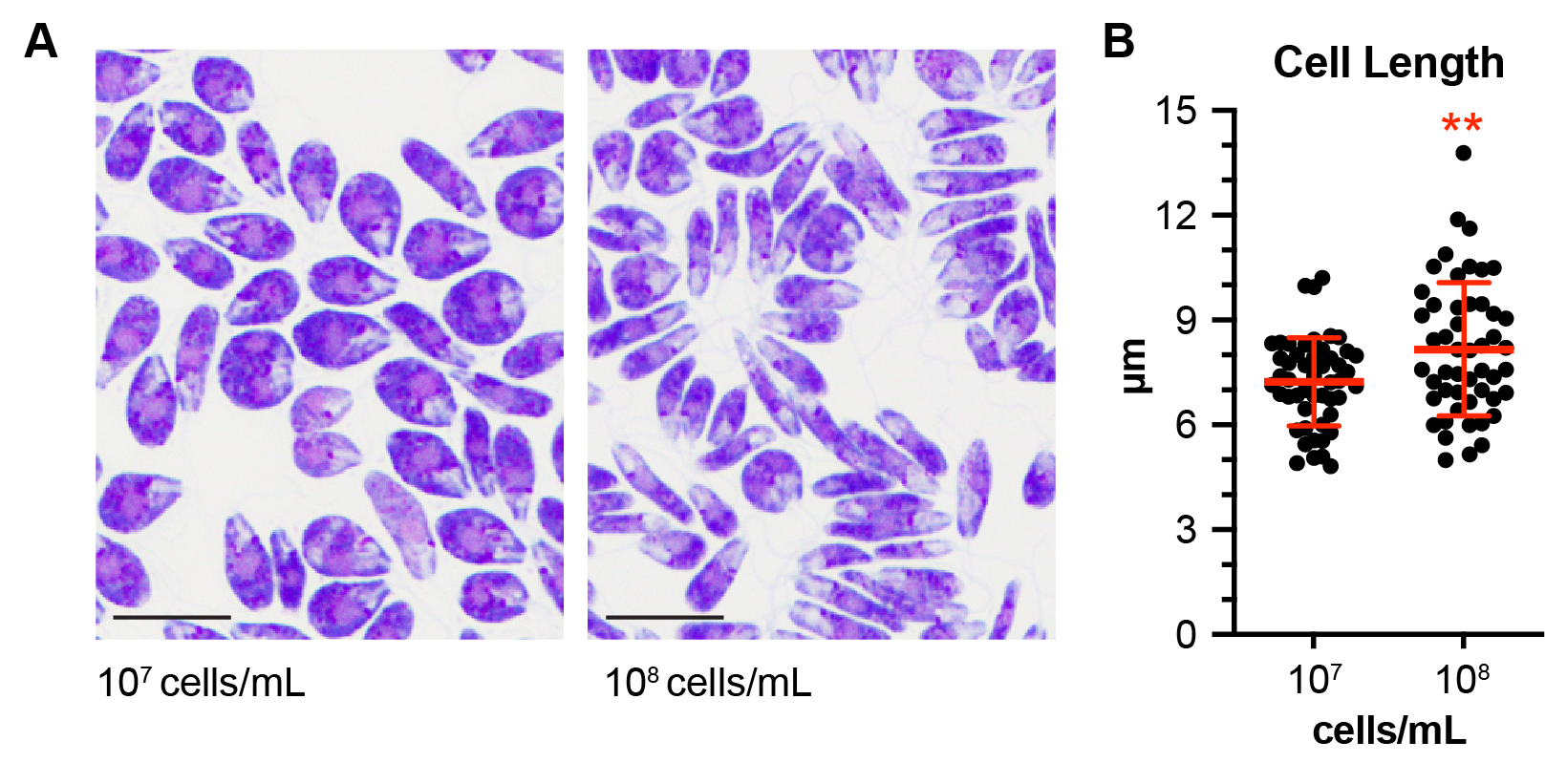
** anterior-posterior axis of the cell body excluding the flagellum. The data are normally distributed (Shapiro-Wilk test). The red line indicates the mean and standard deviation. An unpaired t test indicates the cell length distributions are significantly different with a P value of 0.005.


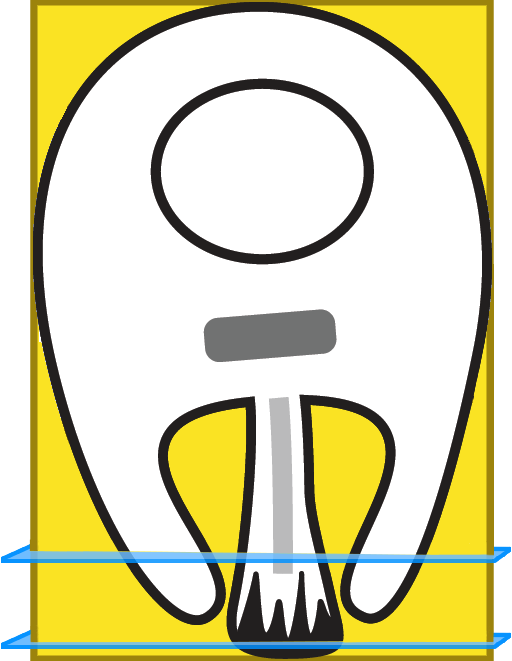


**Figure S2.** Illustration of sectioning planes used for transmission electron microscopy. An adherent cell shown in cross section (yellow plane; sections in Fig 3A-C, and F-G). Sections cut *en face* at different distances from the culture plate (blue planes). Depending on the position of the *en face* section relative to the culture plate, the osmiophilic hemidesmosome-like structure will appear darker (Fig 3E) or lighter (Fig 3D) and other cell features, such as the cell cytoplasm and mitochondrion may be visible (Fig 3D).


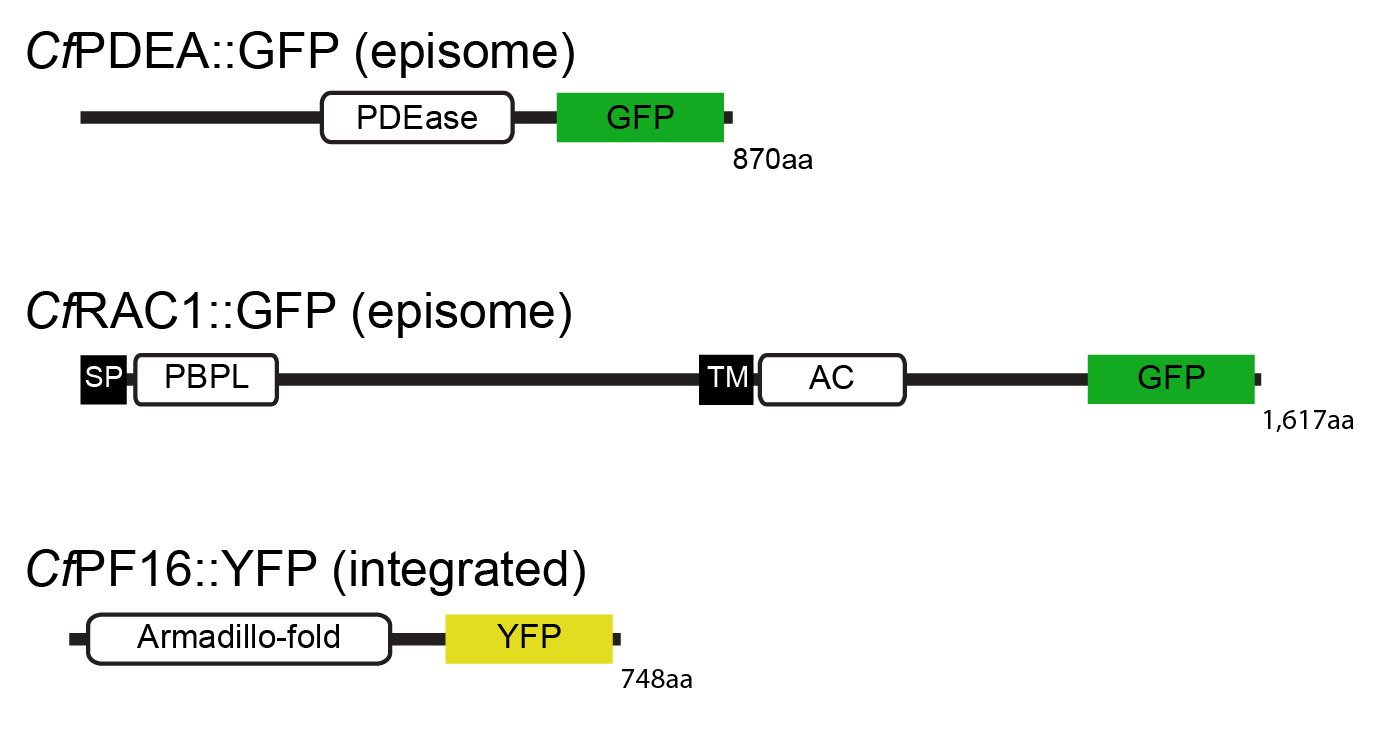


**Figure S3.** Schematic diagram of tagged proteins used for localization studies. Open reading frames encoding *Cf*PDEA (CFAC1_140020300) and *Cf*RAC1 (CFAC1_090006500) were cloned without their C-terminal stop codon into a vector resulting in an in-frame fusion with the coding sequence of GFP (green rectangle). The resulting constructs were transfected into *C. fasciculata* and lines episomally expressing the tagged proteins were selected using hygromycin. A single allele of *Cf*PF16 (CFAC1_170028000) was fused in-frame at its C-terminus with YFP (yellow rectangle) by homologous recombination followed by neomycin selection. The number of amino acids (aa) for each fusion protein is provided. Domains and their relative positions indicated in the different constructs are 3'5'-cyclic nucleotide phosphodiesterase, catalytic domain (PDEase; IPR002073), Periplasmic binding protein-like I (PBPL; IPR028082), Nucleotide cyclase (AC; IPR029787), Armadillo-type fold (Armadillo-fold; IPR016024), signal peptide for secretion (SP), and transmembrane domain (TM). Further details are provided in the Materials and Methods.


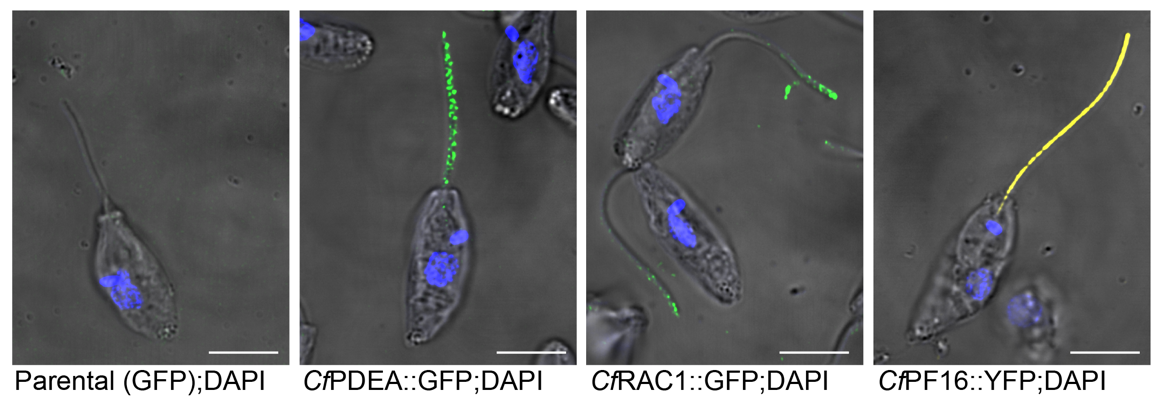


**Figure S4.** Overlay images show the extent of fluorescent signal along the swimming cell flagellum. Overlay of the brightfield and fluorescent images of parental Cf-C1, *Cf*PDEA::GFP, *Cf*RAC1::GFP, and *Cf*PF16::YFP from Figure 6. The fluorescence of *Cf*PDEA::GFP reaches the cell body and weakly inside, similar, but less uniform as *Cf*PF16::YFP. In contrast, *Cf*RAC1::GFP is more restricted to the distal part of the flagellum. Parental cells lack GFP signal. The scale bars are 5 µm.


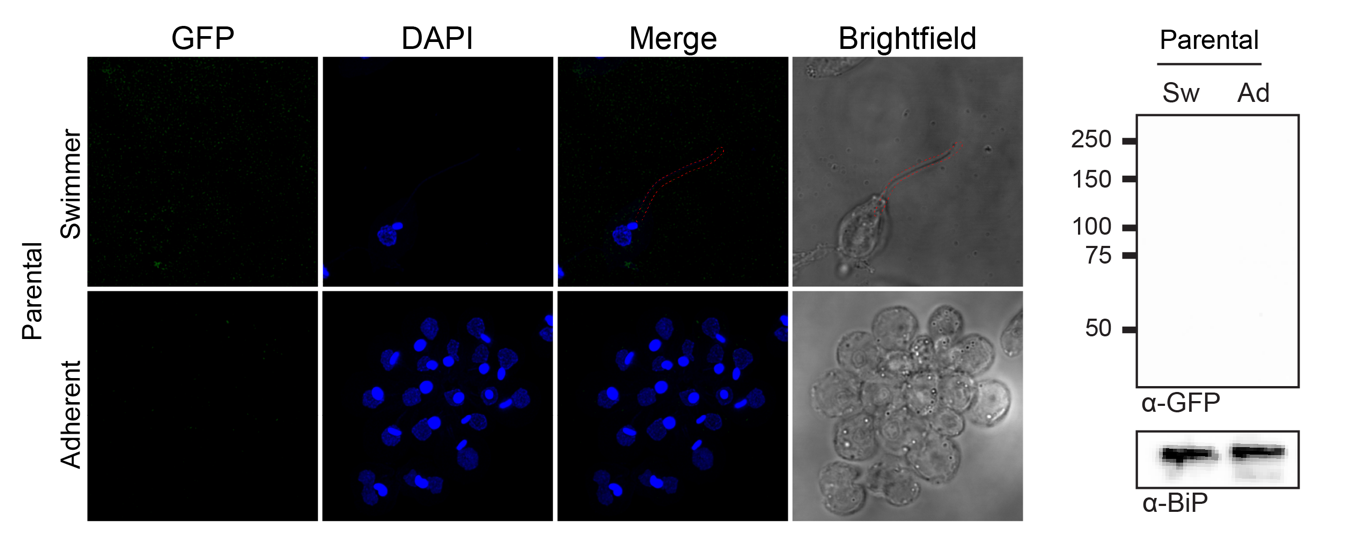


**Figure S5.** Parental Cf-C1 lack GFP signal by confocal and western blot analyses. Deconvolved confocal images of GFP and DAPI images of fixed swimmer and attached cell cultures taken with the same settings as shown in Figure 6. Fluorescent images are shown individually and merged. A brightfield image for each is included for reference and was used to generate the dashed outline of the flagellum in the merged image. A western blot of extracts of swimming and attached cells probed with anti-GFP and anti-BiP (loading control) antibodies.


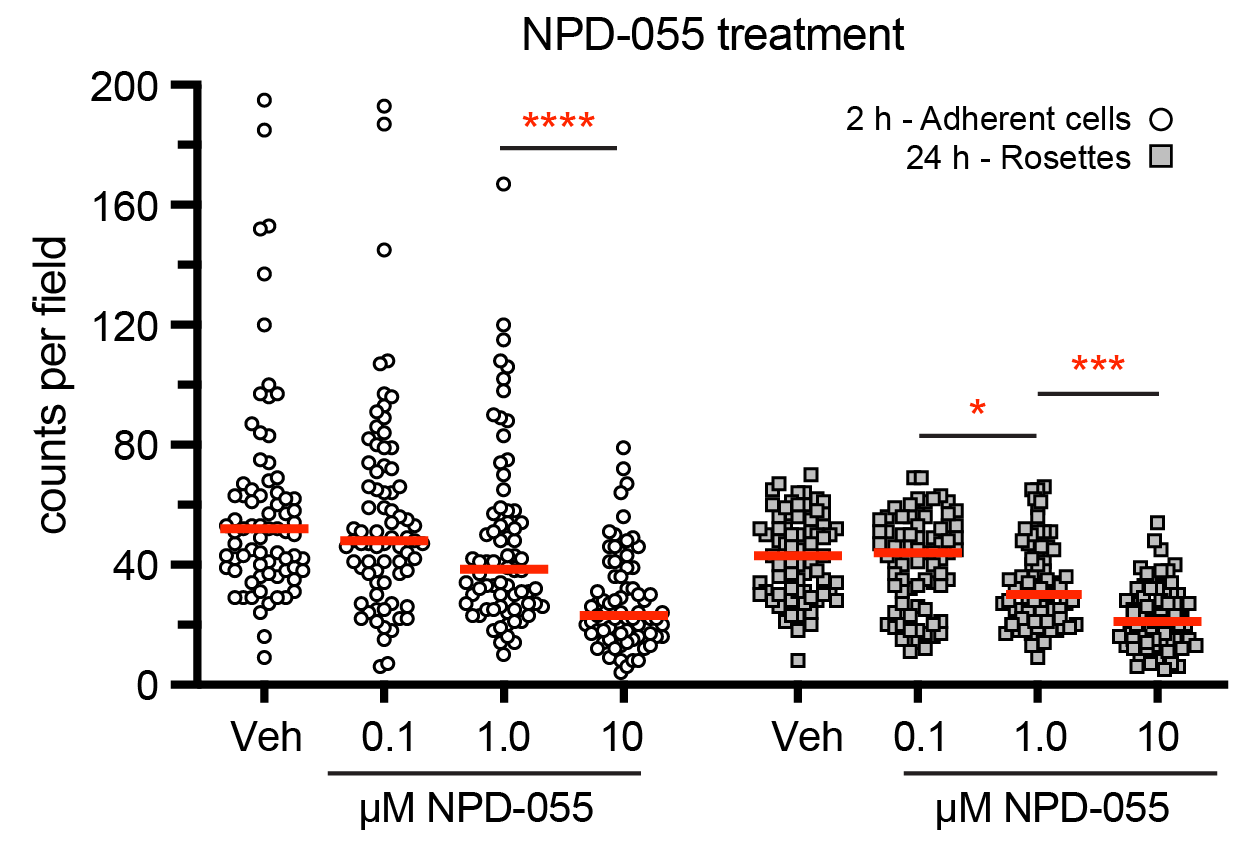


**Figure S6.** Phosphodiesterase inhibitor NPD-055 partially reduces *C. fasciculata* adherence. Adherence assays performed in the presence in the presence of different amounts of NPD-055 compared to vehicle-only treated controls. The number of single adhered cells at 2 hours (open circles) and the number of rosettes (≥ 4 cells, gray squares) at 24 hours were counted and compared to those from a vehicle-treated control sample (Veh). Treatments were present in the media both during the 2-hour plating time and the subsequent 24 hours of culture. Data are not normally distributed. Red lines indicate the median. All comparisons were made, but only the groups that are significantly different from the adjacent treatment are shown. Asterisks indicate Kruskal-Wallis test with Dunn’s correction P<0.0001 (****), P<0.001 (***), and P<0.05 (*).

**Supplementary Table 1.** Proteomics results comparing protein expression levels in adherent (attached) *C. fasciculata* compared to swimming *C. fasciculata* parasites in culture.

**Supplementary Table 2.** Sequences for primers used in this study.

**Video S1.** 4x10^5^ parental Cf-C1 cells were added to wells of a 24-well plate and imaged at 75 second intervals for 16 hours to obtain movies of *C. fasciculata* as they adhere. This video shows the full imaging frame at 60x magnification, which highlights several adhesion events occurring during the imaging time. Note an oblong out-of-focus object appears to the left of center. The scale bar is 50 µm.

**Video S2.** Close up and cropped version of Video S1. For reference, the same oblong out-of-focus object that is visible near the center of Video S1 appears at the left edge of this video. Note when a dividing pair of cells first adheres to the culture dish surface (time 5:42:30), one cell swims away. The other remains characteristically attached at the junction between the cell body and the flagellum. Both the cell body and the flagellum move independently until the flagellum is retracted and the cell body is round (time 8:40:00). The first division as an adherent cell occurs at approximately 9:08:45. One of the adherent daughters divides again approximately 4 hours and 4 minutes later (time 13:12:30). The other adherent daughter divides approximately 5 hours and 25 minutes later (time 14:33:45). The scale bar is 10 µm.
