# Supplementary material for: Adhesion of Crithidia fasciculata promotes a rapid change in developmental fate driven by cAMP signaling
Source: mSphere. 2024 Sep 24;9(10):e00617-24. doi: 10.1128/msphere.00617-24 (PMC11520290; doi:10.1128/msphere.00617-24)
Supplement: Table S2 — Sequences of oligonucleotide primers used to create localization constructs. [file msphere.00617-24-s0003.pdf]

Primer name	Sequence
pLENTeYFP_f	5'-gtgagcaagggcgaggagc-3'
pLENTNeo_r	5'-tcagaagaactcgtcaagaag-3'
PF16cterm500_f	5'-aaggaggcggaggatcata-3'
PF16cterm_r	5'-gctcctcgcccttgctcacgtgctgctgcacgtggtagttc-3'
PF16utr_f	5'-cttcttgacgagttcttctgaaaaaaaaagggaaggagaggcgac-3'
PF16utr500_r	5'-atagtgatgcttgccgtcgt-3'
PF16_500bpnest_f	5'-gctgctgcctggtcact-3'
PF16_500bpnest_r	5'-ccttccttcactgcctctgt-3'
PF16ORF_f	5’-gacgatccagcgtatcaagg-3’
PF16UTR_r	5’-tctttgcattgagcgagcta-3’
CfRAC6500_Nd_f	5’-gcggcgcatatgatgtccccccctctgtggacgc-3’
CfRAC6500_Kp_r	5’-gcggcgggtaccctgcttgcccgaaaatccatt-3’
CfPDEA_Nd_f	5’-tatcatatgatgtccgatttcaaagagcag-3'
CfPDEA_Bg_r	5’-tatagatctcgagtcatcgtggctagcccag-3'
